# Identification of plasma microRNA expression changes in multiple system atrophy and Parkinson’s disease

**DOI:** 10.1186/s13041-019-0471-2

**Published:** 2019-05-14

**Authors:** Hisashi Uwatoko, Yuka Hama, Ikuko Takahashi Iwata, Shinichi Shirai, Masaaki Matsushima, Ichiro Yabe, Jun Utsumi, Hidenao Sasaki

**Affiliations:** Department of Neurology, Faculty of Medicine and Graduate School of Medicine, Hokkaido University, North 15 West 7, Kita-Ku, Sapporo, Hokkaido 060-8368 Japan

**Keywords:** Multiple system atrophy, Parkinson’s disease, microRNA, Plasma, Microarray, Quantitative polymerase chain reaction, Hsa-miR-19b-3p, Hsa-miR-24-3p, Hsa-miR-671-5p

## Abstract

MicroRNAs (miRNAs) are endogenous small (18–25 nt), single-stranded, non-coding RNAs that play key roles in post-transcriptional gene expression regulation. The expression profiles of miRNAs in biofluids and tissues change in various diseases. Multiple system atrophy (MSA) and Parkinson’s disease (PD) are both categorized as α-synucleinopathies and often present with similar clinical manifestations. This study aimed to identify miRNAs that are differently expressed in plasma samples of PD patients, MSA patients, and healthy controls. We used microarray analysis to screen for miRNAs that are up- and down-regulated in these patients and analyzed the relative-quantitative expression levels of the identified miRNAs by reverse transcription quantitative polymerase chain reaction (RT-qPCR). Hsa-miR-671-5p, hsa-miR-19b-3p, and hsa-miR-24-3p showed significantly different expression levels among patients with MSA-C, MSA-P, or PD, and healthy controls. Hsa-miR-671-5p levels were lower in the MSA-P and PD than the MSA-C and control groups, hsa-miR-19b-3p levels were higher in the PD than the other groups, and hsa-miR-24-3p levels were higher in the PD than the MSA-C group. Hsa-miR-671-5p was the first miRNA shown to be expressed differently between MSA-C and MSA-P in plasma. Interestingly, the expression levels of hsa-miR-19b-3p and hsa-miR-24-3p were positively correlated, indicating that these miRNAs may be involved in the same processes in PD pathogenesis. Our findings suggest that hsa-miR-671-5p, hsa-miR-19b-3p, and hsa-miR-24-3p may reflect the pathophysiology or symptoms of PD and MSA.

**Electronic supplementary material:**

The online version of this article (10.1186/s13041-019-0471-2) contains supplementary material, which is available to authorized users.

## Introduction

Multiple system atrophy (MSA) is an adult-onset progressive neurodegenerative disorder that is clinically characterized by autonomic dysfunction, cerebellar ataxia, poorly L-dopa-responsive parkinsonism, and pyramidal dysfunction. Based on the predominant features observed in clinical examinations, MSA is clinically divided into two phenotypes: the Parkinsonian variant (MSA-P) and the cerebellar variant (MSA-C) [1]. Pathological features include the presence of glial cytoplasmic inclusions (GCIs) in oligodendroglia that contains misfolded a-synuclein, neuronal loss, astroglial cytoplasmic inclusions, and gliosis [2]. The degree of neurodegeneration correlates with disease duration and the major clinical phenotype [3]. Parkinsonism is known to correlate with the severity of neuronal loss in the striatonigral structures [4]. In contrast, cerebellar ataxia is strongly correlated with Purkinje cell loss and cell loss in the inferior olives and basis pontis [4].

Parkinson’s disease (PD) is the second most frequent chronic neurodegenerative disorder and is clinically characterized by motor and nonmotor symptoms. The crucial pathological features of PD are the loss of dopaminergic neurons within the substantia nigra pars compacta [5] and the deposition of α-synuclein, which aggregates in a misfolded state and forms intracellular inclusions within the cell body (Lewy bodies) and processes (Lewy neurites) of neurons [6].

MSA and PD are both categorized as α-synucleinopathies and, at the early stage of the disease, often present similar clinical manifestations, which often makes precise differentiation and diagnosis of these conditions difficult. Therefore, the identification of disease biomarkers that can differentiate MSA and PD is strongly desired.

MicroRNAs (miRNAs) are endogenous small (18–25 nt), single-stranded, non-coding RNAs that have been identified as key regulators of gene expression. MiRNAs regulate post-transcriptional gene expression in animals and plants by binding to complementary targets of messenger RNAs (mRNAs), leading to mRNA translational inhibition or degradation, and play a central role in cell differentiation, proliferation, neuronal patterning, and cell survival [7]. There is accumulating evidence suggesting that the dysregulation of miRNAs and their biogenesis machinery is involved in the development of various diseases such as cancers, infections, and cardiovascular disease [8].

The expression profiles of miRNAs in biofluids and tissues have been reported to be altered in various diseases, including cancers, viral infections, diabetes, metabolic disease, cardiovascular disease, and psychiatric disorders. MiRNAs have been proposed to be useful as biomarkers for these conditions [9, 10]. Studies have also suggested that the miRNA expression profiles change in neurological disorders, such as Alzheimer’s disease, amyotrophic lateral sclerosis, PD, MSA, multiple sclerosis, and epilepsy [11–14]. However, only a few studies have described the miRNA expression differences between PD and MSA or between MSA-C and MSA-P.

In order to identify plasma miRNAs that are differently expressed among MSA patients (MSA-C and MSA-P), PD patients, and normal controls, we screened for miRNAs that are up- and down-regulated in MSA patients by using microarray analysis and performed quantitative analyses of the expression levels of the identified miRNAs by reverse transcription quantitative polymerase chain reaction (RT-qPCR).

## Methods

### Patients and sample collection

Healthy controls and patients with probable MSA-C, probable MSA-P, and PD who were diagnosed at Hokkaido University Hospital, Obihiro Kosei General Hospital, and Kushiro Rosai Hospital were enrolled in this study. Diagnosis of MSA-C and MSA-P was made on the basis of the second consensus statement on the diagnosis of MSA [15]. Patient background data (age, sex, disease duration, comorbidities, and clinical parameters) at the time of sample collection were retrospectively obtained from medical records. Clinical parameters included scores on the Scale for the Assessment and Rating of Ataxia (SARA) [16], Barthel Index [17], the Unified Multiple System Atrophy Rating Scale (UMSARS) [18], and the Unified Parkinson’s Disease Rating Scale (UPDRS) [19].

Patients with concurrent malignant tumors, psychiatric disorders, collagen diseases, endocrine diseases, or infections were excluded from our study, since these conditions are previously reported to alter the expression profile of some miRNAs. For the same reason, healthy controls with no known neurological disease or comorbidities were selected. In the MSA-C and MSA-P groups, blood samples that had been obtained at the time of or prior to the diagnosis of probable MSA were used for analyses.

In microarray analysis, plasma samples of 11 patients with probable MSA (eight with MSA-C, three with MSA-P) and of six age- and sex-matched healthy controls fulfilling the criteria listed above were used. In RT-qPCR, plasma samples were obtained from 31 patients with probable MSA-C, 30 with probable MSA-P, and 28 with PD, as well as from 28 healthy controls.

Blood samples were obtained from participants in disodium ethylenediaminotetraacetate (Na_2_EDTA) tubes. Plasma was separated by centrifugation of the blood at 3000×*g* for 30 min at 4 °C immediately after blood sample collection from participants and frozen at − 80 °C until the RNA extraction. We excluded plasma samples with hemolysis or chyle, since these conditions might alter the plasma miRNA profiles [20].

Plasma samples were selected for miRNA analysis, since they are easily and non-invasively obtainable, and since the coagulation process may affect miRNA profiles in serum samples [21].

This study was approved by the Medical Ethics Committee of the Hokkaido University Graduate School of Medicine, and written informed consents were obtained from all participants.

### Microarray analysis

Total RNA was extracted from 300 μL of the frozen plasma using a 3D-Gene® RNA extraction reagent (TORAY Industries, Inc., Tokyo, Japan), and half of the extracted RNA was used for analysis. MiRNAs were labeled using Exiqon miRCURY LNA™ microRNA Array Power Labeling kit (Exiqon Inc., Woburn, MA, USA) and were analyzed with one-channel microarray using the 3D-Gene® Human miRNA oligo chip (Ver. 17.0) (TORAY Industries, Inc., Tokyo, Japan), which can analyze the expression of 1720 miRNAs.

From the output fluorescent signals, we subtracted the background noise and performed calculations using the global normalization method with a median value of 25.

### Reverse transcription quantitative polymerase chain reaction (RT-qPCR)

Total RNA was extracted from frozen plasma (200 μL) using a miRNeasy Serum/Plasma Kit (QIAGEN Inc. Valencia, CA, USA). Plasma samples were thawed on ice and centrifuged at 3000×*g* for 15 min at 4 °C before RNA extraction to thoroughly abate the cellular composition and the buffy coat. As a spike-in control, 400 amol of *Caenorhabditis elegans* miR-39-3p (Cel-miR-39-3p) synthetic oligonucleotide RNA (Sigma-Aldrich, Saint Louis, Missouri, USA) was added to the plasma after addition of denaturing solution. The concentration of the extracted total RNA was measured using Bioanalyzer 2100 with Agilent RNA 6000 pico kit (Agilent Technology Inc. Urdorf, Switzerland); 1 ng of total RNA extracted from plasma was reverse-transcribed into complementary DNA (cDNA) in a 20-μL reaction mixture using miScript II RT kit (QIAGEN Inc. Valencia, CA, USA). Samples that showed irregular RNA peaks or peaks indicative of contamination in electropherograms of Agilent RNA 6000 pico kit were excluded from further analysis. The cDNA was diluted 5×, and 1 μL of the obtained samples was used for qPCR using the miScript SYBR® Green PCR Kit (QIAGEN Inc. Valencia, CA, USA) and miScript primer Assays (QIAGEN Inc. Valencia, CA, USA) for each miRNA (hsa-miR-15b-5p, hsa-miR-19b-3p, hsa-miR-24-3p, hsa-miR-371b-5p, hsa-miR-663a, hsa-miR-671-5p, hsa-miR-920, hsa-miR-3622b-5p, hsa-miR-4708-3p, hsa-miR-4722-5p, and hsa-miR-4736). qPCR was performed in duplicate for each sample using Applied Biosystem® StepOnePlus™ real time PCR system (Applied Biosystems, Foster City, CA, USA), and the expression levels of miRNAs were comparatively quantified using the ΔΔCT method. In the ΔΔCT method, the PCR cycles required to reach threshold (threshold cycle, CT) were measured for each miRNA, and ΔCT was calculated by subtracting the CT value of each miRNA from that of the internal control (hsa-miR-4516) and used for comparison among groups.

### MiRNA target prediction and gene ontology (GO) analysis

In order to search for pathways or processes that are relevant to the up-regulated and down-regulated miRNAs, we extracted predicted target genes of the identified miRNAs using miRmap [22] (mirmap.ezlab.org) and searched for statistically significant GO processes relevant to the predicted genes using MetaCore™ from Clarivate Analytics (Philadelphia, PA, USA).

### Statistical analysis

Data were analyzed using JMP® Version 14 (SAS Institute Inc., Cary, NC, 1987–2007). Kruskal-Wallis test and Welch’s t-test were performed for comparison of miRNA expression levels in each group. Patients’ clinical background data were analyzed using Welch’s t-test. Correlations of miRNA expressions and clinical data were calculated using Spearman’s rho test. *P* values less than 0.05 were considered to be statistically significant.

## Results

### Screening for up- and down-regulated miRNAs in microarray analysis

Background data of the enrolled participants for the microarray analysis are shown in Table 1. The average disease duration of MSA was 2.67 years. No significant differences in sex ratio or age were observed between the MSA and control groups.

**Table 1:** Clinical characteristics of patients with MSA and healthy controls in microarray analysis

	N	Female	Male	Age, yr. (mean ± S.E.)	Disease duration, yr. (mean ± S.E.)	UMSARS (mean ± S.E.)				
						Part 1	Part 2	Part 3 (ΔsBP)	Part 3 (ΔdBP)	Part 4
MSA	13	7 (53.8%)	6 (46.2%)	62.64 ± 1.97	2.67 ± 0.30	18.18 ± 1.45	16.45 ± 2.46	−28.15 ± 7.16	−9.62 ± 4.81	2.36 ± 0.37
Control	6	3 (50.0%)	3 (50.0%)	60.67 ± 0.80	–	–	–	–	–	–

*MSA* multiple system atrophy, *UMSARS* Unified Multiple System Atrophy Rating Scale, *ΔsBP* systolic blood pressure reduction on standing, *ΔdBP* diastolic blood pressure reduction on standing, *S.E.* standard error

Of the 1720 miRNAs detectable on the 3D-Gene® Human miRNA oligo chip (Ver. 17.0), 279 were detected in all samples obtained from both MSA and control groups. Four miRNAs showed significantly higher (up-regulated miRNAs) and 75 miRNAs showed significantly lower normalized values (down-regulated miRNAs) in the MSA than in the control group, as assessed by Welch’s t-test and Kruskal-Wallis test (*p* < 0.05). These miRNAs were selected as candidate miRNAs for the qPCR analysis **(**Additional file 1: Table S1**).**

In order to further narrow down the number of candidate miRNAs for qPCR, we selected down-regulated miRNAs with very strong statistical down-regulation in MSA (*p* < 0.01, Kruskal-Wallis test) and average normalized values higher than 80. The four up-regulated and 13 down-regulated miRNAs that were selected as candidate targets for qPCR are listed in Table 2.

**Table 2:** Candidate miRNAs for qPCR identified in microarray analysis

		Normalized Value (Average ± S.E.)		Fold Change	*p*-value
		MSA	Control	MSA/Control	Kruskal-Wallis test
Up-regulated miRNAs	hsa-miR-371b-5p	126.33 ± 33.77	25.55 ± 1.53	4.945	0.012
	hsa-miR-4708-3p	48.86 ± 11.45	18.78 ± 1.94	2.602	0.010
	hsa-miR-4736	25.26 ± 1.86	15.49 ± 1.65	1.631	0.006
	hsa-miR-663a	1126.09 ± 106.97	775.23 ± 43.51	1.453	0.025
Down-regulated miRNAs	hsa-miR-15b-5p	122.50 ± 25.56	449.37 ± 152.38	0.273	0.010
	hsa-miR-3622b-5p	85.63 ± 26.38	249.39 ± 30.05	0.343	0.008
	hsa-miR-19b-3p	150.93 ± 24.73	431.39 ± 146.55	0.350	0.010
	hsa-miR-920	135.44 ± 27.54	297.63 ± 26.84	0.455	0.008
	hsa-miR-671-5p	172.23 ± 36.23	361.45 ± 63.47	0.476	0.008
	hsa-miR-4722-5p	91.16 ± 10.91	172.25 ± 14.11	0.529	0.003
	hsa-miR-24-3p	482.25 ± 63.88	888.09 ± 44.05	0.543	0.002
	hsa-miR-149-3p	573.56 ± 90.68	1025.11 ± 82.38	0.560	0.008
	hsa-miR-4728-5p	112.43 ± 11.72	189.34 ± 13.59	0.594	0.003
	hsa-miR-3162-5p	155.65 ± 14.27	261.60 ± 29.02	0.595	0.006
	hsa-miR-4270	132.64 ± 17.07	214.49 ± 9.16	0.618	0.006
	hsa-miR-4667-5p	89.86 ± 5.61	144.17 ± 5.63	0.623	0.001
	hsa-miR-4726-5p	116.39 ± 8.44	181.47 ± 7.01	0.641	< 0.001

*MSA* multiple system atrophy, *S.E.* standard error

Among the miRNAs identified in the microarray analysis, we selected all four up-regulated miRNAs (hsa-miR-371b-5p, hsa-miR-4708-3p, hsa-miR-4736, and hsa-miR-663a) and the top seven down-regulated miRNAs (hsa-miR-15b-5p, hsa-miR-3622b-5p, hsa-miR-19b-3p, hsa-miR-920, hsa-miR-671-5p, hsa-miR-4722-5p, and hsa-miR-24-3p), according to the fold change, and performed qPCR. Hsa-miR-4516 was selected as the internal control, since it was equally expressed in the MSA and control groups and showed sufficient levels and low standard error in the microarray analysis. Hsa-miR-4516 also showed small dispersion and equivalency in patients with amyotrophic lateral sclerosis and healthy controls and thus, was selected as an internal control in our previous research [12].

### Quantitative analysis of miRNA expression with RT-qPCR

Background data of the enrolled participants for the RT-qPCR analysis are shown in Table 3. Sex distribution was equal in all groups, but patients in the MSA-C and control groups were significantly younger than patients in the MSA-P and PD groups (*p* < 0.05). Patients in the MSA-C and MSA-P groups showed similar disease duration, whereas those in the PD group showed a significantly longer disease duration (*p* < 0.05). The scores for UMSARS parts 1, 2, and 4 were slightly but significantly higher in the MSA-P than the MSA-C group (*p* < 0.05), while Barthel Index was significantly lower than that in the PD group (*p* < 0.05).

**Table 3:** Clinical characteristics of MSA patients and healthy controls in qPCR

	n	Female	Male	Age, yr. (mean ± S.E.)	Disease Duration, yr. (mean ± S.E.)	Clinical Scale (mean ± S.E.)				
						UMSARS				
						Part 1	Part 2	Part 3 (ΔsBP)	Part 3 (ΔdBP)	Part 4
MSA-C	31	16 (51.6%)	15 (48.4%)	60.72 ± 1.73	3.09 ± 0.32	16.83 ± 1.62	15.90 ± 1.69	−28.93 ± 4.25	−12.33 ± 2.62	2.04 ± 0.20
MSA-P	30	17 (56.7%)	13 (43.3%)	68.08 ± 1.31 ^a^	3.01 ± 0.32	19.63 ± 1.69	22.30 ± 1.85	−20.56 ± 3.23	−10.33 ± 2.50	2.88 ± 0.19
						UPDRS				
						Part 1	Part 2	Part 3	Part 4	
PD	28	15 (53.6%)	13 (46.4%)	68.97 ± 1.32 ^a^	9.58 ± 0.98 ^b^	2.64 ± 0.42	12.78 ± 1.84	25.32 ± 3.40	2.12 ± 0.51	
Control	28	13 (46.4%)	15 (53.6%)	63.18 ± 1.29						

*MSA* multiple system atrophy, *PD* Parkinson’s disease, *UMSARS* Unified Multiple System Atrophy Rating Scale, *ΔsBP* systolic blood pressure reduction on standing, *ΔdBP* diastolic blood pressure reduction on standing, *S.E.* standard error

^a^ Patients in the MSA-P and PD groups were significantly older than those in the MSA-C and control groups (p < 0.05)

^b^ Patients with PD had significantly longer disease duration compared to those with MSA-C and MSA-P (*p* < 0.05)

Of the 11 targets, five miRNAs (hsa-miR-371b-5p, hsa-miR-4708-3p, hsa-miR-3622b-5p, hsa-miR-920, and hsa-miR-4722-5p) were excluded from analysis due to primer dimer formation and amplification failure. The average CT value of the spike-in control (cel-miR-39-3p) was 23.66 with a low standard error (S.E.) of 0.18, indicating the equality of miRNA quantity in each sample analyzed in the qPCR.

**Fig. 1:**
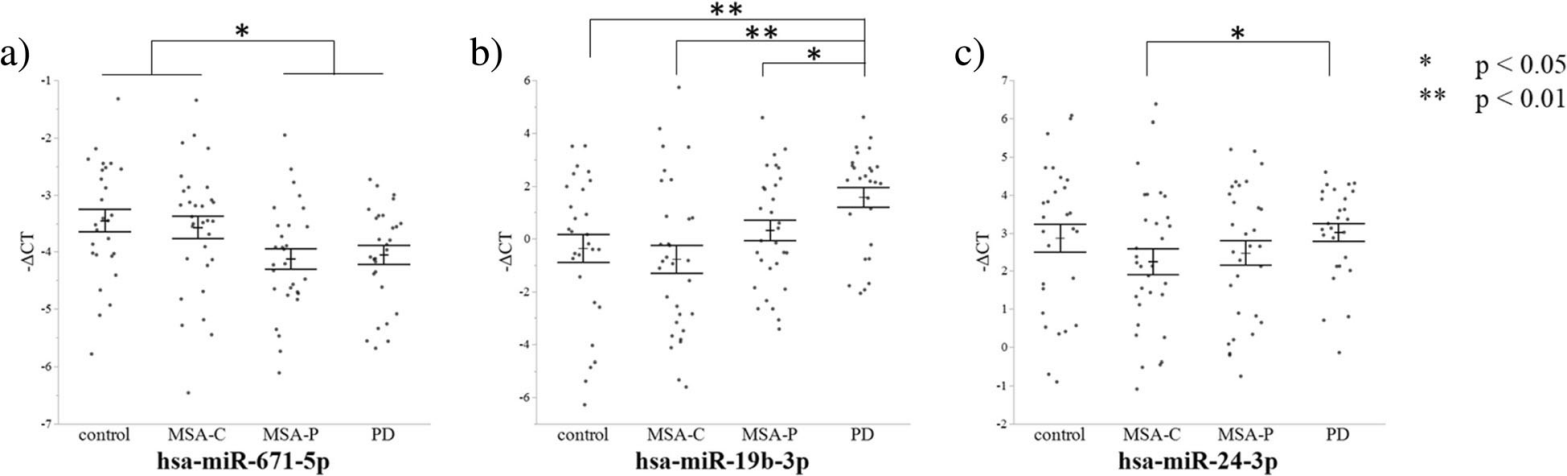
ΔCT values for the control, MSA-C, MSA-P, and PD groups in qPCR. qPCR analysis of miRNAs in plasma of control subjects and of patients with MSA-C, MSA-P and PD revealed **a**) hsa-miR-671-5p down-regulations in the MSA-P and PD groups compared to the control and MSA-C groups, **b**) hsa-miR-19b-3p up-regulation in the PD group compared to other groups, and **c**) hsa-miR-24-3p up-regulations in the PD group compared to the MSA-C group. ΔCT was calculated by subtracting the Threshold Cycle (CT) value of each target miRNA from the CT value of the internal control (hsa-miR-4516). Statistical analyses of differences among groups were performed using Kruskal-Wallis test. *p < 0.05; **p < 0.01

Hsa-miR-671-5p expression levels in the PD group were significantly lower than those in the control (*p* = 0.0280) and MSA-C groups (*p* = 0.0459). Moreover, the expression levels of hsa-miR-671-5p were significantly lower in the MSA-P group than in the control (*p* = 0.0146) and MSA-C groups (*p* = 0.0187) (Fig. 1a). Hsa-miR-19b-3p expression was significantly higher in the PD group than in the control (*p* = 0.0080), MSA-C (*p* = 0.0015), and MSA-P (*p* = 0.0247) groups (Fig. 1b). The PD group also showed a significantly higher level of hsa-miR-24-3p compared to the MSA-C group (*p* = 0.0381) (Fig. 1c). Among miRNAs that were properly amplified by qPCR, the expression levels of hsa-miR-15b-5p, hsa-miR-663a, and hsa-miR-4736 showed no significant intergroup differences (Additional file 5: Figure S1a-c).

In qPCR, no miRNAs were differently expressed between the healthy control group and the MSA-C or MSA group (MSA-C and MSA-P groups combined). Correlation analysis of the miRNA expression with the clinical data (age, sex, disease duration, comorbidities and clinical parameters) revealed no correlation for any of the miRNAs.

**Fig. 2:**
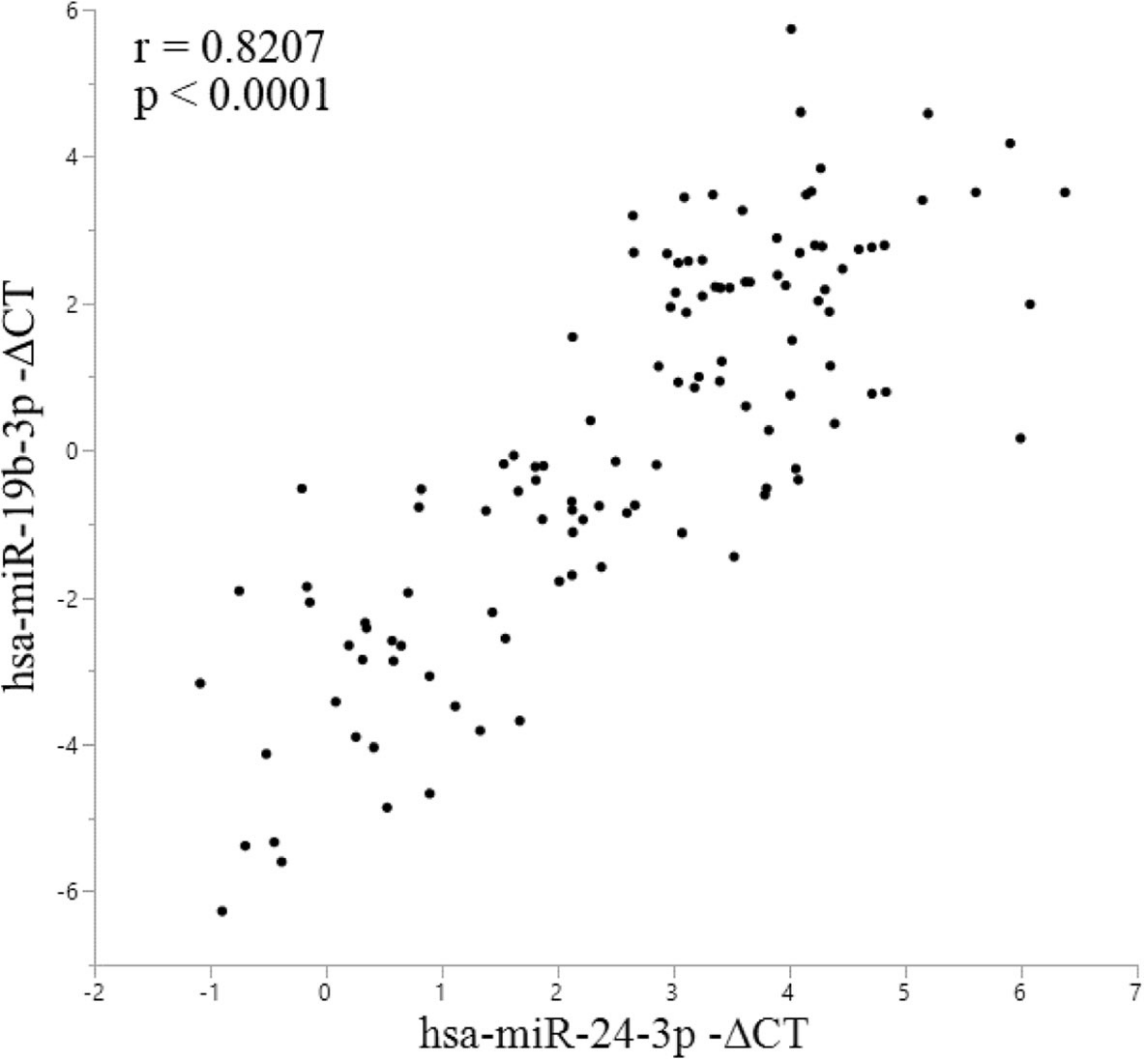
Correlation of hsa-miR-19b-3p and hsa-miR-24-3p expression. Correlation analysis of the expression levels of miRNAs in all plasma samples (*n* = 117) analyzed by qPCR revealed a strong correlation between hsa-miR-19b-3p and hsa-miR-24-3p expression levels was found. The X axis presents the -ΔCT values of hsa-miR-24-3p, and the Y axis presents the -ΔCT value of hsa-miR-19b-3p. ΔCT was calculated by subtracting the CT value of each target miRNA from the CT value of the internal control (hsa-miR-4516). Data were analyzed using Spearman’s rho test

Interestingly, correlation analysis among the expression level of each miRNA revealed a positive correlation (r = 0.8207, *p* < 0.0001) between hsa-miR-19b-3p and hsa-miR-24-3p expression levels (Fig. 2).

Since age was significantly different among groups, and previous studies have reported miRNA profile differences among different age groups [23, 24], we performed subgroup analysis of patients aged 50 to 75 years. This analysis showed a down-regulation of hsa-miR-671-5p in patients with MSA-P (*p* = 0.0410) and PD (*p* = 0.0359) compared to healthy controls. In this subgroup analysis, hsa-miR-19b-3p expression was also higher in patients with PD than in control subjects (*p* < 0.05), patients with MSA-C (*p* < 0.05), and those with MSA-P (*p* < 0.05), whereas different expression levels of hsa-miR-24-3p were not observed, presumably due to the smaller sample size (Additional file 6: Figure S2a-c).

### miRNA target gene prediction and GO analysis

The top 50 predicted targets of hsa-miR-671-5p, hsa-miR-19b-3p, and hsa-miR-24-3p were obtained using miRmap (Additional file 2: Table S2). The top 15 statistically significant GO processes relevant to the top 50 predicted hsa-miR-671-5p target genes included multiple processes related to muscle contraction, neuron differentiation, and nervous system development (Table 4). Similarly, the top 15 statistically significant GO processes relevant to the top 50 predicted hsa-miR-19b-3p and hsa-miR-24-3p target genes are respectively shown in Additional file 3: Table S3 and Additional file 4: Table S4.

**Table 4:** Top 15 statistically significant GO processes relevant to the top 50 predicted target genes of hsa-miR-671-5p

	GO processes	*p*-value	FDR
1	regulation of vascular smooth muscle contraction	5.47E-12	1.95E-08
2	positive regulation of smooth muscle contraction	7.63E-11	1.36E-07
3	positive regulation of muscle contraction	1.69E-09	2.01E-06
4	regulation of neuron differentiation	3.71E-09	2.38E-06
5	positive regulation of neuron differentiation	3.75E-09	2.38E-06
6	nervous system development	4.14E-09	2.38E-06
7	phospholipase C-activating G-protein coupled acetylcholine receptor signaling pathway	4.67E-09	2.38E-06
8	regulation of system process	6.77E-09	2.77E-06
9	saliva secretion	7.77E-09	2.77E-06
10	adenylate cyclase-inhibiting G-protein coupled acetylcholine receptor signaling pathway	7.77E-09	2.77E-06
11	secretion by tissue	1.43E-08	4.55E-06
12	regulation of blood circulation	1.53E-08	4.55E-06
13	regulation of muscle contraction	2.27E-08	5.66E-06
14	negative regulation of catecholamine secretion	2.33E-08	5.66E-06
15	regulation of smooth muscle contraction	2.39E-08	5.66E-06

*GO* gene ontology, *FDR* false discovery rate

Although not included in the top 50 predicted target genes, *FMR1* (miRmap score: 94.89), *LRRK2* (miRmap score: 84.55)*,* and *COQ2* (miRmap score: 42.68) were included in the complete list of predicted hsa-miR-19b-3p target genes; *HIP1R* (miRmap score: 98.20)*, ATP13A2* (miRmap score: 85.30)*, SYT11* (miRmap score: 79.01)*,* and *RAB39B* (miRmap score: 78.96) were included in predicted hsa-miR-24-3p target genes; and *CHCHD2* (miRmap score: 93.34), *LRRK2* (miRmap score: 95.15), *PLA2G6* (miRmap score: 79.87), *EDN1* (miRmap score: 90.26), and *SNCA* (miRmap score: 71.44) were included in the hsa-miR-671-5p target genes.

Since the expression levels both of hsa-miR-19b-3p and hsa-miR-24-3p were up-regulated in the PD group and showed strong correlation, we searched for GO processes that are similarly relevant to the top 100 predicted target genes of these miRNAs and selected the top 15 GO processes. The identified GO processes included processes related to dopamine and catecholamine uptake involved in synaptic transmission and processes related to regulation of dopamine secretion (Table 5).

**Table 5:** Top 15 GO processes similarly relevant to the top 100 predicted target genes of hsa-miR-19b-3p and hsa-miR-24-3p

	GO processes	err(−log(*p*-Value))	miR-19b-3p		miR-24-3p	
			*p*-value	FDR	*p*-value	FDR
1	cation transport	8.91E-03	1.95E-01	2.62E-01	2.01E-01	2.99E-01
2	organonitrogen compound catabolic process	1.13E-02	4.91E-01	5.33E-01	4.99E-01	5.63E-01
3	developmental process involved in reproduction	1.13E-02	5.35E-02	1.02E-01	5.01E-02	1.34E-01
4	nitrogen compound transport	1.27E-02	5.37E-03	1.80E-02	4.70E-03	4.43E-02
5	Golgi reassembly	2.03E-02	2.43E-02	5.67E-02	2.08E-02	8.85E-02
6	positive regulation of glomerular filtration	2.13E-02	2.91E-02	6.45E-02	2.49E-02	9.58E-02
7	regulation of systemic arterial blood pressure by vasopressin	2.30E-02	3.86E-02	7.98E-02	3.31E-02	1.11E-01
8	operant conditioning	2.30E-02	3.86E-02	7.98E-02	3.31E-02	1.11E-01
9	regulation of dopamine uptake involved in synaptic transmission	2.32E-02	1.53E-03	6.62E-03	1.12E-03	2.07E-02
10	regulation of catecholamine uptake involved in synaptic transmission	2.32E-02	1.53E-03	6.62E-03	1.12E-03	2.07E-02
11	platelet dense granule organization	2.38E-02	4.33E-02	8.70E-02	3.72E-02	1.18E-01
12	negative regulation of dopamine secretion	2.38E-02	4.33E-02	8.70E-02	3.72E-02	1.18E-01
13	intestine smooth muscle contraction	2.45E-02	4.80E-02	9.42E-02	4.12E-02	1.23E-01
14	interleukin-1 beta secretion	2.45E-02	4.80E-02	9.42E-02	4.12E-02	1.23E-01
15	mast cell chemotaxis	2.45E-02	4.80E-02	9.42E-02	4.12E-02	1.23E-01

*GO* gene ontology, *FDR* false discovery rate

## Discussion

Using microarray analysis, we screened for plasma miRNAs that were differentially expressed in patients with MSA and in healthy controls and then performed qPCR to quantitatively analyze the top four up-regulated and seven down-regulated miRNAs. In qPCR analysis, hsa-miR-671-5p expression was significantly lower in the MSA-P and PD groups than in the MSA-C and healthy groups; in contrast, hsa-miR-19b-3p expression was significantly higher in the PD group than in the other groups, and hsa-miR-24-3p expression was significantly higher in the PD than in the MSA-C group. Furthermore, statistically significant positive correlations between the expression levels of hsa-miR-19b-3p and those of hsa-miR-24-3p were observed. GO processes related to the predicted target genes of these miRNAs included processes associated with muscle contraction, neuron differentiation, nervous system development, synaptic transmission, and dopamine secretion.

Also, the complete lists of predicted target genes of hsa-miR-19b-3p, hsa-miR-24-3p, and hsa-miR-671-5p included *FMR1*, *LRRK2*, *COQ2*, *HIP1R*, *ATP13A2*, *SYT11*, *RAB39B*, *CHCHD2*, *PLA6G2*, *EDN1*, *and SNCA*, all of which have been previously reported to be associated with PD and/or MSA [25–28].

Expression-level changes of these miRNAs are thought to be solely due to MSA or PD, since we omitted concurrent diseases that may alter miRNA expression levels.

### Hsa-miR-671-5p

The microarray analysis showed hsa-miR-671-5p down-regulation in the MSA group. Hsa-miR-671-5p was significantly down-regulated in the MSA-P and PD groups compared to the other groups in qPCR. In contrast, hsa-miR-671-5p down-regulation in the MSA-C or MSA group (MSA-C and MSA-P combined) was not observed in qPCR. There are a few reports on hsa-miR-671-5p expression changes in breast cancer, glioblastoma multiforme, Kawasaki’s disease, and pediatric chordomas [29–32], but no study has reported hsa-miR-671-5p down-regulation in neurodegenerative disorders.

GO analysis using MetaCore™ revealed processes related to muscle contraction and neuron differentiation. Considering these GO processes and previous studies reporting that exercise and physical activity alter the expression of some miRNAs [33, 34], one possible hypothesis for the down-regulation of plasma hsa-miR-671-5p in the MSA-P and PD groups is that the expression of this miRNA may reflect the altered physical activity due to Parkinsonian symptoms or changes in muscle tone, which is known to increase in Parkinsonism [35]. Since little is known about the expression change and function of hsa-miR-671-5p, more analyses of its expression in other samples (serum, cerebrospinal fluid, brain tissue etc.) in various diseases, including other neurodegenerative diseases, are needed for further understanding the hsa-miR-671-5p expression profile.

Furthermore, to our knowledge, hsa-miR-671-5p is the first miRNA identified to be differently expressed between MSA-C and MSA-P using RT-qPCR. Although regarded as a single entity, MSA-C and MSA-P have multiple different characteristics. Epidemiologically, the MSA-P subtype outnumbers the MSA-C subtype in western countries [36, 37], whereas the latter is dominant in Japan [38–40]. The brain pathology in MSA-P shows dominant neurodegeneration in the basal ganglia, while in MSA-C, neurodegeneration is predominantly observed in the ponto-cerebello-olivary system and cerebellum [41]. The radiological findings for MSA-C and MSA-P are also different [42]. Considering these multiple different clinical, pathological, and epidemiological features between the two subtypes of MSA, there is a high possibility that miRNA expression profiles also differ between MSA-C and MSA-P. Therefore, further studies on the miRNA expression differences between MSA-C and MSA-P may be important for understanding MSA.

### Hsa-miR-19b-3p and hsa-miR-24-3p

Hsa-miR-19b-3p and hsa-miR-24-3p were found to be down-regulated in the MSA group compared to healthy controls in the microarray analysis. Although we could not validate the down-regulation of these miRNAs in the MSA-C or MSA-P groups by qPCR, hsa-miR-19b-3p in the PD group showed significant up-regulation compared to the other groups, and hsa-miR-24-3p in the PD group showed significant up-regulation compared to the MSA-C group.

Previous studies have also described expression-level changes in hsa-miR-19b-3p and hsa-miR-24-3p in patients with PD and MSA. Vallelunga et al. reported the up-regulation of hsa-miR-24 in the serum of patients with PD and MSA using TaqMan LowDensity Array technology and RT-qPCR by TaqMan method [43]. Botta-Orfila et al. found a down-regulation of hsa-miR-19b-3p in the serum of idiopathic PD patients and PD patients with LRRK2 G2019S mutation, also using TaqMan LowDensity Array technology and RT-qPCR (TaqMan) [44]. Marques et al. performed analysis of miRNA expression in the cerebrospinal fluid using TaqMan MicroRNA primer and found a down-regulation of hsa-miR-19b-3p in patients with MSA and of hsa-miR-24-3p in patients with PD and MSA [45].

Although the changes in hsa-miR-19b-3p and hsa-miR-24-3p expression levels in our study partially contradict the results of previous studies, the finding that hsa-miR-19b-3p and hsa-miR-24-3p are differently expressed in various biofluids of PD and MSA patients is of importance. Considering such changes, the strong correlation of hsa-miR-19b-3p and hsa-miR-24-3p expression levels in the current study, as well as the predicted GO processes, including those relevant to synaptic transmission and dopamine secretion, changes in the expression of these miRNAs might reflect the pathogenesis of or the pathological changes in PD.

In conclusion, plasma hsa-miR-671-5p, hsa-miR-19b-3p, and hsa-miR-24-3p were found to be differently expressed in MSA and PD. These miRNAs may become markers that reflect the pathophysiology or symptoms of PD and MSA. Further analyses of miRNAs in larger cohorts and in various samples are required for further understanding of MSA and PD and for establishing useful clinical biomarkers.

## Additional files

Additional file 1: **Table S1.** miRNAs with significantly higher and lower expression compared to healthy controls in microarray analysis. (DOCX 38 kb)

Additional file 2: **Table S2.** Top 50 predicted target genes for each miRNA using miRmap. (DOCX 22 kb)

Additional file 3: **Table S3.** Top 15 statistically significant GO processes relevant to the top 50 predicted target genes of hsa-miR-19b-3p. (DOCX 18 kb)

Additional file 4: **Table S4.** Top 15 statistically significant GO processes relevant to the top 50 predicted target genes of hsa-miR-24-3p. (DOCX 18 kb)

Additional file 5: **Figure S1.** ΔCT values for the control, MSA-C, MSA-P, and PD groups in qPCR. qPCR analysis of miRNAs in the plasma of control participants and patients with MSA-C, MSA-P, and PD revealed no statistically significant expression differences for a) hsa-miR-15b-5p, b) hsa-miR-663a, and c) hsa-miR-4736 among the control, MSA-C, MSA-P, and PD groups. (DOCX 80 kb)

Additional file 6: **Figure S2.** ΔCT values for the control, MSA-C, MSA-P, and PD groups in qPCR for patients aged between 50 and 75 years. qPCR analysis of miRNAs in plasma samples from patients with MSA-C (*n* = 23), MSA-P (*n* = 24), and PD (*n* = 20), and from healthy controls (*n* = 28) aged between 50 and 75 years. a) hsa-miR-671-5p was down-regulated in the MSA-P and PD groups compared to the control group, b) hsa-miR-19b-3p was up-regulated in the PD group compared to the other groups, c) hsa-miR-24-3p was similarly expressed in all groups in patients aged between 50 and 75 years. Statistical analysis of each group was performed using Kruskal-Wallis test. *p < 0.05; **p < 0.01. (DOCX 87 kb)

## Abbreviations

GO: Gene ontology
miRNA: microRNA
MSA: Multiple system atrophy
PD: Parkinson’s disease
RT-qPCR: Reverse transcription quantitative polymerase chain reaction
SARA: The Scale for the Assessment and Rating of Ataxia
UMSARS: The Unified Multiple System Atrophy Rating Scale
UPDRS: The Unified Parkinson’s Disease Rating Scale
