# Supplementary material for: Identification of plasma microRNA expression changes in multiple system atrophy and Parkinson’s disease
Source: Mol Brain. 2019 May 14;12:49. doi: 10.1186/s13041-019-0471-2 (PMC6518614; doi:10.1186/s13041-019-0471-2)
Supplement: Supplementary file 1 — Table S1. miRNAs with significantly higher and lower expression compared to healthy controls in microarray analysis. (DOCX 38 kb) [file 13041_2019_471_MOESM1_ESM.docx]

| **Additional Table 1.** miRNAs with significantly higher and lower expression in patients with MSA than in healthy controls in microarray analysis | | | | | | | | | | | | | |  |
| --- | --- | --- | --- | --- | --- | --- | --- | --- | --- | --- | --- | --- | --- | --- |
|  |  |  | Normalized Fluorescent Signal (Average ± S.E.) | | | | | | | |  | *p*-value | |  |
|  |  |  | MSA | | | | Control | | | | Fold Change | Welch's t-test | Kruskal-Wallis test |  |
| Up-regulated miRNAs |  | hsa-miR-4736 | 25.26 | ± | 1.86 |  | 15.49 | ± | 1.65 |  | 1.631 | 0.005 | 0.006 |  |
|  |  | hsa-miR-4708-3p | 48.86 | ± | 11.45 |  | 18.78 | ± | 1.94 |  | 2.602 | 0.020 | 0.010 |  |
|  |  | hsa-miR-371b-5p | 126.33 | ± | 33.77 |  | 25.55 | ± | 1.53 |  | 4.945 | 0.025 | 0.012 |  |
|  |  | hsa-miR-663a | 1126.09 | ± | 106.97 |  | 775.23 | ± | 43.51 |  | 1.453 | 0.046 | 0.025 |  |
|  |  |  |  |  |  |  |  |  |  |  |  |  |  |  |
| Down-regulated miRNAs |  | hsa-miR-4726-5p | 116.39 | ± | 8.44 |  | 181.47 | ± | 7.01 |  | 0.641 | <0.001 | <0.001 |  |
|  |  | hsa-miR-4667-5p | 89.86 | ± | 5.61 |  | 144.17 | ± | 5.63 |  | 0.623 | <0.001 | 0.001 |  |
|  |  | hsa-miR-24-3p | 482.25 | ± | 63.88 |  | 888.09 | ± | 44.05 |  | 0.543 | 0.003 | 0.002 |  |
|  |  | hsa-miR-370-3p | 46.36 | ± | 4.07 |  | 73.51 | ± | 4.30 |  | 0.631 | 0.002 | 0.003 |  |
|  |  | hsa-miR-4728-5p | 112.43 | ± | 11.72 |  | 189.34 | ± | 13.59 |  | 0.594 | 0.006 | 0.003 |  |
|  |  | hsa-miR-2392 | 38.02 | ± | 3.28 |  | 64.60 | ± | 4.54 |  | 0.589 | 0.002 | 0.003 |  |
|  |  | hsa-miR-4489 | 37.05 | ± | 3.18 |  | 59.99 | ± | 3.15 |  | 0.618 | 0.001 | 0.003 |  |
|  |  | hsa-miR-4722-5p | 91.16 | ± | 10.91 |  | 172.25 | ± | 14.11 |  | 0.529 | 0.001 | 0.003 |  |
|  |  | hsa-miR-3150a-3p | 36.78 | ± | 6.82 |  | 81.57 | ± | 8.78 |  | 0.451 | 0.003 | 0.004 |  |
|  |  | hsa-miR-658 | 74.19 | ± | 7.21 |  | 112.67 | ± | 4.13 |  | 0.658 | 0.007 | 0.004 |  |
|  |  | hsa-miR-1587 | 55.52 | ± | 6.61 |  | 86.59 | ± | 2.33 |  | 0.641 | 0.019 | 0.004 |  |
|  |  | hsa-miR-4270 | 132.64 | ± | 17.07 |  | 214.49 | ± | 9.16 |  | 0.618 | 0.021 | 0.006 |  |
|  |  | hsa-miR-3162-5p | 155.65 | ± | 14.27 |  | 261.60 | ± | 29.02 |  | 0.595 | 0.003 | 0.006 |  |
|  |  | hsa-miR-4428 | 50.96 | ± | 5.76 |  | 89.64 | ± | 8.86 |  | 0.568 | 0.006 | 0.006 |  |
|  |  | hsa-miR-4800-5p | 40.85 | ± | 2.21 |  | 59.57 | ± | 5.11 |  | 0.686 | 0.001 | 0.008 |  |
|  |  | hsa-miR-4776-5p | 29.58 | ± | 3.03 |  | 50.77 | ± | 4.62 |  | 0.583 | 0.003 | 0.008 |  |
|  |  | hsa-miR-149-3p | 573.56 | ± | 90.68 |  | 1025.11 | ± | 82.38 |  | 0.560 | 0.015 | 0.008 |  |
|  |  | hsa-miR-671-5p | 172.23 | ± | 36.23 |  | 361.45 | ± | 63.47 |  | 0.476 | 0.012 | 0.008 |  |
|  |  | hsa-miR-920 | 135.44 | ± | 27.54 |  | 297.63 | ± | 26.84 |  | 0.455 | 0.004 | 0.008 |  |
|  |  | hsa-miR-3187-5p | 63.18 | ± | 18.67 |  | 178.88 | ± | 19.30 |  | 0.353 | 0.003 | 0.008 |  |
|  |  | hsa-miR-3622b-5p | 85.63 | ± | 26.38 |  | 249.39 | ± | 30.05 |  | 0.343 | 0.003 | 0.008 |  |
|  |  | hsa-miR-4648 | 21.24 | ± | 3.06 |  | 36.85 | ± | 3.85 |  | 0.577 | 0.015 | 0.010 |  |
|  |  | hsa-miR-25-3p | 74.52 | ± | 8.22 |  | 152.15 | ± | 30.44 |  | 0.490 | 0.007 | 0.010 |  |
|  |  | hsa-miR-19b-3p | 150.93 | ± | 24.73 |  | 431.39 | ± | 146.55 |  | 0.350 | 0.004 | 0.010 |  |
|  |  | hsa-miR-15b-5p | 122.50 | ± | 25.56 |  | 449.37 | ± | 152.38 |  | 0.273 | 0.003 | 0.010 |  |
|  |  | hsa-miR-642b-3p | 371.48 | ± | 38.25 |  | 525.90 | ± | 15.59 |  | 0.706 | 0.024 | 0.012 |  |
|  |  | hsa-miR-4743-5p | 29.20 | ± | 2.19 |  | 42.06 | ± | 3.90 |  | 0.694 | 0.012 | 0.012 |  |
|  |  | hsa-miR-4721 | 37.37 | ± | 4.49 |  | 61.22 | ± | 5.47 |  | 0.610 | 0.012 | 0.012 |  |
|  |  | hsa-miR-4429 | 141.83 | ± | 10.25 |  | 233.81 | ± | 36.14 |  | 0.607 | 0.004 | 0.012 |  |
|  |  | hsa-miR-4539 | 37.76 | ± | 5.19 |  | 62.46 | ± | 3.96 |  | 0.605 | 0.017 | 0.012 |  |
|  |  | hsa-miR-3917 | 118.63 | ± | 13.90 |  | 199.26 | ± | 21.54 |  | 0.595 | 0.009 | 0.012 |  |
|  |  | hsa-miR-1224-5p | 43.56 | ± | 5.72 |  | 75.02 | ± | 6.40 |  | 0.581 | 0.006 | 0.012 |  |
|  |  | hsa-miR-4259 | 106.21 | ± | 15.01 |  | 190.95 | ± | 23.92 |  | 0.556 | 0.013 | 0.012 |  |
|  |  | hsa-miR-125a-3p | 25.68 | ± | 2.31 |  | 51.74 | ± | 9.46 |  | 0.496 | 0.003 | 0.012 |  |
|  |  | hsa-miR-4513 | 113.19 | ± | 28.88 |  | 264.85 | ± | 28.38 |  | 0.427 | 0.004 | 0.012 |  |
|  |  | hsa-miR-4664-5p | 31.17 | ± | 4.04 |  | 73.35 | ± | 15.26 |  | 0.425 | 0.002 | 0.012 |  |
|  |  | hsa-miR-4751 | 54.98 | ± | 4.10 |  | 75.72 | ± | 6.17 |  | 0.726 | 0.021 | 0.016 |  |
|  |  | hsa-miR-3187-3p | 30.08 | ± | 3.42 |  | 49.37 | ± | 5.05 |  | 0.609 | 0.010 | 0.016 |  |
|  |  | hsa-miR-320d | 73.80 | ± | 11.04 |  | 165.46 | ± | 40.27 |  | 0.446 | 0.012 | 0.016 |  |
|  |  | hsa-miR-4738-3p | 28.15 | ± | 1.78 |  | 36.17 | ± | 2.48 |  | 0.778 | 0.032 | 0.020 |  |
|  |  | hsa-miR-4697-5p | 143.68 | ± | 16.37 |  | 204.99 | ± | 12.14 |  | 0.701 | 0.031 | 0.020 |  |
|  |  | hsa-miR-4689 | 551.63 | ± | 68.45 |  | 798.15 | ± | 16.20 |  | 0.691 | 0.043 | 0.020 |  |
|  |  | hsa-miR-4430 | 46.35 | ± | 5.42 |  | 69.94 | ± | 4.47 |  | 0.663 | 0.022 | 0.020 |  |
|  |  | hsa-miR-4750-5p | 64.14 | ± | 8.77 |  | 96.84 | ± | 5.51 |  | 0.662 | 0.032 | 0.020 |  |
|  |  | hsa-miR-1913 | 50.59 | ± | 7.81 |  | 78.40 | ± | 4.50 |  | 0.645 | 0.042 | 0.020 |  |
|  |  | hsa-miR-4538 | 35.64 | ± | 4.03 |  | 55.27 | ± | 4.40 |  | 0.645 | 0.014 | 0.020 |  |
|  |  | hsa-miR-4440 | 30.79 | ± | 5.32 |  | 50.11 | ± | 1.89 |  | 0.614 | 0.026 | 0.020 |  |
|  |  | hsa-miR-3147 | 69.91 | ± | 11.98 |  | 130.45 | ± | 12.53 |  | 0.536 | 0.009 | 0.020 |  |
|  |  | hsa-miR-16-5p | 231.41 | ± | 42.84 |  | 577.33 | ± | 152.48 |  | 0.401 | 0.011 | 0.020 |  |
|  |  | hsa-miR-23b-3p | 347.32 | ± | 56.00 |  | 981.74 | ± | 283.08 |  | 0.354 | 0.005 | 0.020 |  |
|  |  | hsa-miR-4690-5p | 67.41 | ± | 6.27 |  | 93.56 | ± | 4.00 |  | 0.721 | 0.017 | 0.025 |  |
|  |  | hsa-miR-4514 | 49.74 | ± | 4.85 |  | 75.50 | ± | 8.13 |  | 0.659 | 0.021 | 0.025 |  |
|  |  | hsa-miR-3154 | 112.90 | ± | 20.77 |  | 207.38 | ± | 17.19 |  | 0.544 | 0.010 | 0.025 |  |
|  |  | hsa-miR-21-5p | 128.82 | ± | 27.74 |  | 442.96 | ± | 158.49 |  | 0.291 | 0.014 | 0.025 |  |
|  |  | hsa-miR-3188 | 44.16 | ± | 4.15 |  | 57.57 | ± | 2.12 |  | 0.767 | 0.040 | 0.032 |  |
|  |  | hsa-miR-4442 | 578.32 | ± | 61.53 |  | 837.89 | ± | 98.44 |  | 0.690 | 0.040 | 0.032 |  |
|  |  | hsa-miR-3925-5p | 68.02 | ± | 7.62 |  | 99.56 | ± | 8.51 |  | 0.683 | 0.046 | 0.032 |  |
|  |  | hsa-miR-1307-3p | 42.72 | ± | 5.42 |  | 67.32 | ± | 6.17 |  | 0.635 | 0.035 | 0.032 |  |
|  |  | hsa-miR-92b-3p | 143.58 | ± | 18.66 |  | 226.65 | ± | 31.78 |  | 0.633 | 0.020 | 0.032 |  |
|  |  | hsa-miR-22-3p | 401.22 | ± | 75.74 |  | 1006.32 | ± | 277.49 |  | 0.399 | 0.013 | 0.032 |  |
|  |  | hsa-miR-99b-5p | 17.05 | ± | 1.06 |  | 25.39 | ± | 3.31 |  | 0.672 | 0.012 | 0.039 |  |
|  |  | hsa-miR-505-5p | 26.69 | ± | 2.72 |  | 41.16 | ± | 5.45 |  | 0.649 | 0.021 | 0.039 |  |
|  |  | hsa-miR-4447 | 109.90 | ± | 14.37 |  | 170.82 | ± | 22.97 |  | 0.643 | 0.035 | 0.039 |  |
|  |  | hsa-miR-106b-5p | 77.33 | ± | 11.40 |  | 181.30 | ± | 66.91 |  | 0.427 | 0.043 | 0.039 |  |
|  |  | hsa-miR-23a-3p | 456.71 | ± | 84.67 |  | 1112.09 | ± | 309.73 |  | 0.411 | 0.016 | 0.039 |  |
|  |  | hsa-miR-425-5p | 63.25 | ± | 10.10 |  | 159.93 | ± | 45.69 |  | 0.396 | 0.013 | 0.039 |  |
|  |  | hsa-let-7f-5p | 48.14 | ± | 9.04 |  | 198.85 | ± | 73.41 |  | 0.242 | 0.011 | 0.039 |  |
|  |  | hsa-miR-3619-3p | 273.52 | ± | 14.01 |  | 345.30 | ± | 28.76 |  | 0.792 | 0.035 | 0.049 |  |
|  |  | hsa-miR-3622a-5p | 62.77 | ± | 6.42 |  | 86.61 | ± | 6.20 |  | 0.725 | 0.039 | 0.049 |  |
|  |  | hsa-miR-498-5p | 28.71 | ± | 2.76 |  | 41.83 | ± | 4.72 |  | 0.686 | 0.029 | 0.049 |  |
|  |  | hsa-miR-4496 | 61.33 | ± | 11.01 |  | 103.32 | ± | 10.37 |  | 0.594 | 0.030 | 0.049 |  |
|  |  | hsa-miR-4723-5p | 940.35 | ± | 187.32 |  | 1686.66 | ± | 374.42 |  | 0.558 | 0.034 | 0.049 |  |
|  |  | hsa-miR-29a-3p | 38.52 | ± | 4.81 |  | 76.39 | ± | 18.49 |  | 0.504 | 0.017 | 0.049 |  |
|  |  | hsa-miR-4454 | 2843.95 | ± | 374.88 |  | 6868.69 | ± | 1994.39 |  | 0.414 | 0.012 | 0.049 |  |
|  |  | hsa-miR-26a-5p | 283.84 | ± | 58.67 |  | 946.85 | ± | 339.54 |  | 0.300 | 0.022 | 0.049 |  |

MSA, multiple system atrophy； S.E., standard error.
