# Supplementary material for: Identification of plasma microRNA expression changes in multiple system atrophy and Parkinson’s disease
Source: Mol Brain. 2019 May 14;12:49. doi: 10.1186/s13041-019-0471-2 (PMC6518614; doi:10.1186/s13041-019-0471-2)
Supplement: Supplementary file 2 — Table S2. Top 50 predicted target genes for each miRNA using miRmap. (DOCX 22 kb) [file 13041_2019_471_MOESM2_ESM.docx]

**Additional table 2.** Top 50 predicted target genes for each miRNA using miRmap

|  | hsa-miR-19b-3p | |  | hsa-miR-24-3p | |  | hsa-miR-671-5p | |
| --- | --- | --- | --- | --- | --- | --- | --- | --- |
|  | Gene | miRmap score |  | Gene | miRmap score |  | Gene | miRmap score |
| 1 | *ATXN1* | 99.657 |  | *BOK* | 99.981 |  | *MDM4* | 99.916 |
| 2 | *RPS6KA5* | 99.546 |  | *ORAI2* | 99.977 |  | *ITSN1* | 99.883 |
| 3 | *CDS1* | 99.387 |  | *FTO* | 99.949 |  | *SLC30A6* | 99.879 |
| 4 | *CLOCK* | 99.364 |  | *FOXK1* | 99.858 |  | *PTPRT* | 99.864 |
| 5 | *ZMYND11* | 99.270 |  | *VPS53* | 99.842 |  | *CRAMP1L* | 99.851 |
| 6 | *SECISBP2L* | 99.203 |  | *TAOK1* | 99.841 |  | *MR1* | 99.800 |
| 7 | *SLC30A7* | 99.199 |  | *PGPEP1* | 99.839 |  | *SSR1* | 99.773 |
| 8 | *TNRC6B* | 99.191 |  | *ENTPD1* | 99.836 |  | *DLGAP2* | 99.746 |
| 9 | *MDM4* | 99.187 |  | *MTHFR* | 99.821 |  | *USP46* | 99.728 |
| 10 | *AFF1* | 99.070 |  | *SOGA1* | 99.817 |  | *C1orf21* | 99.701 |
| 11 | *CHIC1* | 99.032 |  | *LRRC20* | 99.776 |  | *EHD3* | 99.677 |
| 12 | *TSC1* | 99.030 |  | *AP3S2* | 99.770 |  | *C10orf25* | 99.670 |
| 13 | *STOX2* | 98.990 |  | *DRAXIN* | 99.770 |  | *SATB2* | 99.666 |
| 14 | *CAND1* | 98.809 |  | *CHST4* | 99.759 |  | *PI4K2A* | 99.660 |
| 15 | *ACSL4* | 98.798 |  | *KCNJ5* | 99.752 |  | *SYPL2* | 99.646 |
| 16 | *SKIDA1* | 98.623 |  | *MLEC* | 99.744 |  | *CAMK1D* | 99.624 |
| 17 | *LRP2* | 98.610 |  | *LRPAP1* | 99.697 |  | *PACSIN1* | 99.594 |
| 18 | *PMEPA1* | 98.562 |  | *RAB3B* | 99.682 |  | *ADRA2B* | 99.572 |
| 19 | *ARHGEF26* | 98.520 |  | *RRP8* | 99.678 |  | *BCL9L* | 99.557 |
| 20 | *FICD* | 98.376 |  | *PRIMA1* | 99.664 |  | *LIX1L* | 99.557 |
| 21 | *EXOC5* | 98.355 |  | *ITSN1* | 99.641 |  | *DVL3* | 99.546 |
| 22 | *ENPP5* | 98.322 |  | *TMEM154* | 99.640 |  | *ANKH* | 99.545 |
| 23 | *KLF7* | 98.292 |  | *TMEM178B* | 99.621 |  | *PPM1L* | 99.533 |
| 24 | *ZNF217* | 98.270 |  | *FAM168B* | 99.610 |  | *SLC6A11* | 99.519 |
| 25 | *CEP350* | 98.247 |  | *hsa-mir-4763* | 99.593 |  | *SLIT3* | 99.515 |
| 26 | *ZNF677* | 98.189 |  | *IBA57* | 99.469 |  | *CPNE2* | 99.481 |
| 27 | *LGALSL* | 98.175 |  | *NFASC* | 99.467 |  | *SLC1A2* | 99.480 |
| 28 | *TRIM23* | 98.119 |  | *AVL9* | 99.465 |  | *GALNT10* | 99.478 |
| 29 | *DSEL* | 98.113 |  | *NDST1* | 99.444 |  | *FGD6* | 99.443 |
| 30 | *SYNM* | 98.091 |  | *GINS2* | 99.436 |  | *XPR1* | 99.431 |
| 31 | *PRUNE2* | 97.991 |  | *PPARGC1B* | 99.432 |  | *SPTB* | 99.428 |
| 32 | *CYLD* | 97.958 |  | *DNAJB2* | 99.420 |  | *PSMB2* | 99.422 |
| 33 | *HBP1* | 97.909 |  | *PHF15* | 99.418 |  | *CCDC6* | 99.404 |
| 34 | *CREB3L2* | 97.865 |  | *SUSD2* | 99.415 |  | *KCNA6* | 99.395 |
| 35 | *GJA1* | 97.825 |  | *SCAMP5* | 99.398 |  | *SMAD3* | 99.384 |
| 36 | *ZNF831* | 97.805 |  | *LIMD1* | 99.392 |  | *LIN9* | 99.376 |
| 37 | *ABCC3* | 97.801 |  | *ZBTB44* | 99.376 |  | *GPR107* | 99.366 |
| 38 | *DNAJC16* | 97.789 |  | *RAB3IP* | 99.349 |  | *C16orf72* | 99.346 |
| 39 | *RBBP8* | 97.779 |  | *CDC42EP3* | 99.348 |  | *CFL2* | 99.340 |
| 40 | *ZNF238* | 97.752 |  | *PDXK* | 99.347 |  | *SYT2* | 99.339 |
| 41 | *ZC3HAV1L* | 97.744 |  | *SH3PXD2A* | 99.344 |  | *XCR1* | 99.320 |
| 42 | *KBTBD8* | 97.730 |  | *CCDC93* | 99.341 |  | *KRT9* | 99.311 |
| 43 | *ANKRD13C* | 97.689 |  | *GNE* | 99.329 |  | *EIF4EBP2* | 99.279 |
| 44 | *KCNA4* | 97.676 |  | *KLHL3* | 99.297 |  | *CHRM3* | 99.264 |
| 45 | *SYT1* | 97.659 |  | *C1orf21* | 99.286 |  | *DCAF17* | 99.258 |
| 46 | *SYT6* | 97.657 |  | *NLN* | 99.283 |  | *SYT9* | 99.252 |
| 47 | *BEND4* | 97.642 |  | *GRAMD1B* | 99.264 |  | *PBX1* | 99.228 |
| 48 | *FEM1C* | 97.632 |  | *PLXNA4* | 99.251 |  | *SLC8A2* | 99.204 |
| 49 | *TRIM2* | 97.577 |  | *TMEM213* | 99.247 |  | *OTUB2* | 99.199 |
| 50 | *BMPR2* | 97.535 |  | *IFFO2* | 99.241 |  | *GREM2* | 99.192 |
