# Supplementary material for: Identification of plasma microRNA expression changes in multiple system atrophy and Parkinson’s disease
Source: Mol Brain. 2019 May 14;12:49. doi: 10.1186/s13041-019-0471-2 (PMC6518614; doi:10.1186/s13041-019-0471-2)
Supplement: Supplementary file 3 — Table S3. Top 15 statistically significant GO processes relevant to the top 50 predicted target genes of hsa-miR-19b-3p. (DOCX 18 kb) [file 13041_2019_471_MOESM3_ESM.docx]

| **Additional Table 3.** Top 15 statistically significant gene ontology (GO) processes relevant to the top 50 predicted target genes of hsa-miR-19b-3p | | | |  |
| --- | --- | --- | --- | --- |
|  | GO processes | p-value | FDR |  |
| 1 | negative regulation of cellular macromolecule biosynthetic process | 1.723E-07 | 3.817E-04 |  |
| 2 | negative regulation of macromolecule biosynthetic process | 3.867E-07 | 4.283E-04 |  |
| 3 | negative regulation of cellular biosynthetic process | 9.213E-07 | 6.802E-04 |  |
| 4 | negative regulation of biosynthetic process | 1.384E-06 | 7.666E-04 |  |
| 5 | regulation of macromolecule biosynthetic process | 3.251E-06 | 1.085E-03 |  |
| 6 | regulation of biosynthetic process | 3.921E-06 | 1.085E-03 |  |
| 7 | histone H2A-S1 phosphorylation | 4.408E-06 | 1.085E-03 |  |
| 8 | negative regulation of prostaglandin secretion | 4.408E-06 | 1.085E-03 |  |
| 9 | fast, calcium ion-dependent exocytosis of neurotransmitter | 4.408E-06 | 1.085E-03 |  |
| 10 | regulation of cellular macromolecule biosynthetic process | 7.074E-06 | 1.567E-03 |  |
| 11 | regulation of cellular biosynthetic process | 9.620E-06 | 1.937E-03 |  |
| 12 | negative regulation of nucleobase-containing compound metabolic process | 1.411E-05 | 2.605E-03 |  |
| 13 | negative regulation of nitrogen compound metabolic process | 2.230E-05 | 3.800E-03 |  |
| 14 | acrosomal vesicle exocytosis | 2.638E-05 | 3.895E-03 |  |
| 15 | histone H3-S28 phosphorylation | 2.638E-05 | 3.895E-03 |  |
|  | FDR, false discovery rate |  |  |  |
