# Supplementary material for: Identification of plasma microRNA expression changes in multiple system atrophy and Parkinson’s disease
Source: Mol Brain. 2019 May 14;12:49. doi: 10.1186/s13041-019-0471-2 (PMC6518614; doi:10.1186/s13041-019-0471-2)
Supplement: Supplementary file 4 — Table S4. Top 15 statistically significant GO processes relevant to the top 50 predicted target genes of hsa-miR-24-3p. (DOCX 18 kb) [file 13041_2019_471_MOESM4_ESM.docx]

| **Additional Table 4.** Top 15 statistically significant gene ontology (GO) processes relevant to the top 50 predicted target genes of hsa-miR-24-3p | | | |  |
| --- | --- | --- | --- | --- |
|  | GO processes | p-value | FDR |  |
| 1 | protein sulfation | 6.213E-07 | 9.450E-04 |  |
| 2 | N-acetylglucosamine metabolic process | 8.303E-06 | 6.314E-03 |  |
| 3 | sulfation | 1.466E-05 | 7.432E-03 |  |
| 4 | peptidyl-cysteine methylation | 3.885E-05 | 1.107E-02 |  |
| 5 | glucosamine-containing compound metabolic process | 4.274E-05 | 1.107E-02 |  |
| 6 | positive regulation of dopamine uptake involved in synaptic transmission | 5.820E-05 | 1.107E-02 |  |
| 7 | regulation of vesicle size | 5.820E-05 | 1.107E-02 |  |
| 8 | positive regulation of catecholamine uptake involved in synaptic transmission | 5.820E-05 | 1.107E-02 |  |
| 9 | amino sugar metabolic process | 1.920E-04 | 2.973E-02 |  |
| 10 | vesicle-mediated transport | 2.389E-04 | 2.973E-02 |  |
| 11 | regulation of dopamine uptake involved in synaptic transmission | 2.541E-04 | 2.973E-02 |  |
| 12 | positive regulation of neurotransmitter uptake | 2.541E-04 | 2.973E-02 |  |
| 13 | regulation of catecholamine uptake involved in synaptic transmission | 2.541E-04 | 2.973E-02 |  |
| 14 | positive regulation of synaptic transmission, dopaminergic | 4.028E-04 | 4.376E-02 |  |
| 15 | vesicle-mediated transport in synapse | 4.858E-04 | 4.926E-02 |  |
|  | FDR, false discovery rate |  |  |  |
