# Supplementary figures and images for: Identification of plasma microRNA expression changes in multiple system atrophy and Parkinson’s disease
Source: Mol Brain. 2019 May 14;12:49. doi: 10.1186/s13041-019-0471-2 (PMC6518614; doi:10.1186/s13041-019-0471-2)

**Additional Fig. 1** -ΔCT values for the control, MSA-C, MSA-P, and PD groups in qPCR.


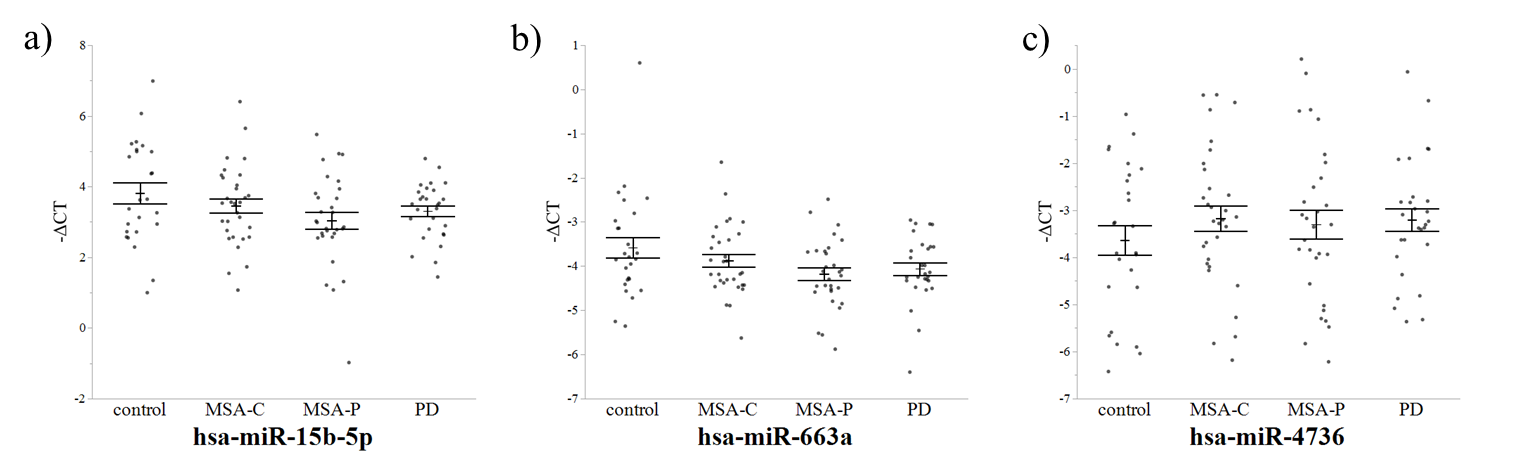

Supplement: Supplementary file 5 — Figure S1. ΔCT values for the control, MSA-C, MSA-P, and PD groups in qPCR. qPCR analysis of miRNAs in the plasma of control participants and patients with MSA-C, MSA-P, and PD revealed no statistically significant expression differences for a) hsa-miR-15b-5p, b) hsa-miR-663a, and c) hsa-miR-4736 among the control, MSA-C, MSA-P, and PD groups. (DOCX 80 kb) [file 13041_2019_471_MOESM5_ESM.docx]
