# Supplementary material for: Identification of plasma microRNA expression changes in multiple system atrophy and Parkinson’s disease
Source: Mol Brain. 2019 May 14;12:49. doi: 10.1186/s13041-019-0471-2 (PMC6518614; doi:10.1186/s13041-019-0471-2)

**Additional Fig. 2** -ΔCT values for the control, MSA-C, MSA-P, and PD groups in qPCR for patients aged between 50 and 75 years.


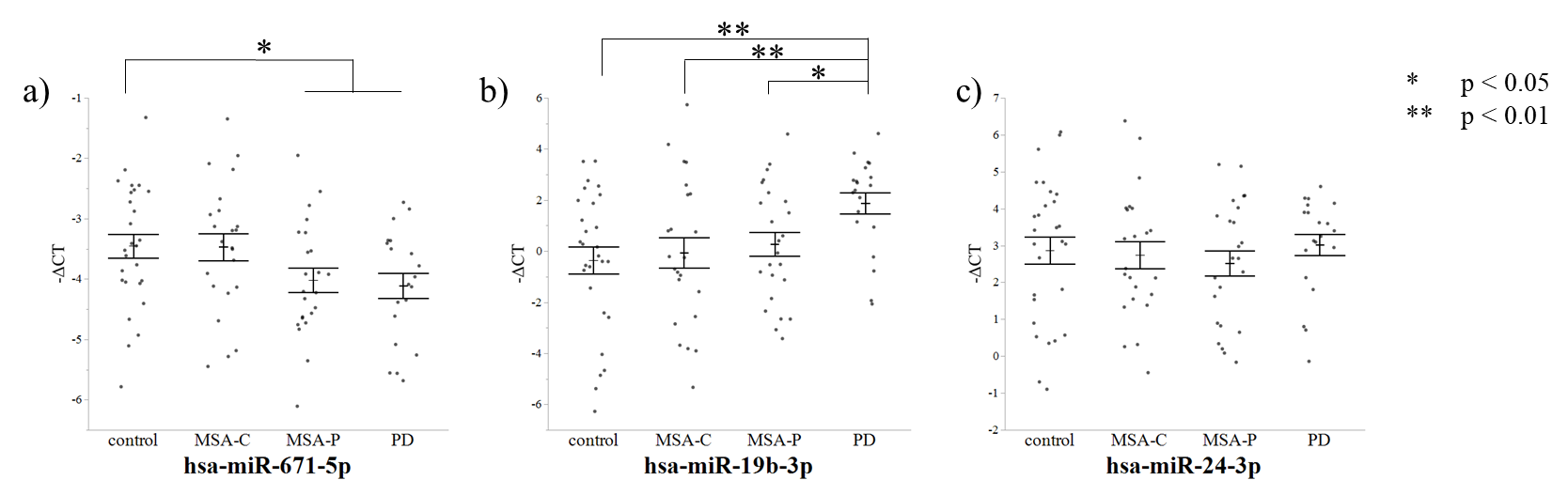

Supplement: Supplementary file 6 — Figure S2. ΔCT values for the control, MSA-C, MSA-P, and PD groups in qPCR for patients aged between 50 and 75 years. qPCR analysis of miRNAs in plasma samples from patients with MSA-C (n = 23), MSA-P (n = 24), and PD (n = 20), and from healthy controls (n = 28) aged between 50 and 75 years. a) hsa-miR-671-5p was down-regulated in the MSA-P and PD groups compared to the control group, b) hsa-miR-19b-3p was up-regulated in the PD group compared to the other groups, c) hsa-miR-24-3p was similarly expressed in all groups in patients aged between 50 and 75 years. Statistical analysis of each group was performed using Kruskal-Wallis test. *p < 0.05; **p < 0.01. (DOCX 87 kb) [file 13041_2019_471_MOESM6_ESM.docx]
